# An Update on Alopecia and its Association With Thyroid Autoimmune Diseases

**DOI:** 10.17925/EE.2023.19.2.10

**Published:** 2023-08-08

**Authors:** Doaa Aboalola, Sihem Aouabdi, Majed Ramadan, Tariq Alghamdi, Mona Alsolami, Dalal Malibari, Rawiah Alsiary

**Affiliations:** 1. King Abdullah International Medical Research Center, Jeddah, Western Region, Saudi Arabia; 2. King Saud bin Abdulaziz University for Health Sciences, Jeddah, Western Region, Saudi Arabia; 3. Ministry of National Guard Health Affairs, Jeddah, Western Region, Saudi Arabia

**Keywords:** Alopecia, autoimmune thyroiditis, focused review, thyroid cancer, thyroid autoimmune diseases and alopecia, thyroid cancer and alopecia

## Abstract

Alopecia is comorbid with several illnesses, including various autoimmune conditions such as thyroid disease. Leukocyte-mediated inflammation of hair follicles in alopecia was first described over a century ago. However, the high prevalence of the role of thyroid autoimmune disease in the pathogenesis of alopecia has only recently come to light, together with a strong association between the two. Therefore, this review focuses on articles published between 2011 and 2022 on alopecia's association with thyroid autoimmune disease, and the mechanism behind it. In addition, it highlights the link between alopecia and thyroid cancer, as patients with alopecia have increased risk of thyroid cancer. In conclusion, this comprehensive, focused, scoping review will serve as a reference highlighting recent information on alopecia, exploring its association with thyroid autoimmune diseases.

Alopecia is a dermatological disorder characterized by hair loss from the scalp or body.^1–3^ It is one of the most common dermatological disorders worldwide and has several aetiologies, such as hereditary background, hormonal imbalance, infection or idiopathic causes.^1,3,4^ Alopecia can be classified into two main categories: scarring (or cicatricial) alopecia and non-scarring (non-cicatricial) alopecia (*Figure 1*).^1–3^

Scarring alopecia is a complex and heterogeneous group of hair disorders resulting in irreversible destruction of the hair follicle, which is then replaced by fibrous scar tissue, due to permanent follicular stem cell damage.^1–3^ Non-scarring alopecia is caused by an alteration in the capillary cycle, which causes temporary or partial damage to hair follicles, resulting in hair shedding followed by hair regrowth.^1,3^

This comprehensive, focused review aims to serve as an up-to-date reference highlighting recent information on alopecia and its association with thyroid autoimmune diseases (TADs), focusing on case-control, retrospective, cross-sectional or cohort designs, and focused review articles published between 2011 and 2022.

## Scarring alopecia

Primary cicatricial alopecia, often termed scarring alopecia, describes a group of hair loss disorders in which the hair follicle is permanently destroyed and replaced by fibrous tissue.^1–3,5–7^ Most types of primary scarring alopecia, some of which overlap or occur simultaneously, are classified as inflammatory skin disorders.^8^ Primary scarring alopecia is less common than non-scarring alopecia, representing about 5–7% of cases.^3,6^ With scarring alopecia, the skin is bald, smooth and shiny inside the patches, and pores are absent due to a complete loss of follicular openings.^6^

The hypotheses proposed to explain why scarring alopecia occurs include the loss of follicular immune privilege, alterations in the microbiota of the local pilosebaceous unit, abnormal lipid metabolism, the possible involvement of mast cells and an association with skin care products and sunscreens.^8–11^ The role of genetics in scarring alopecia also remains unclear.^8,9^

**Figure 1:**
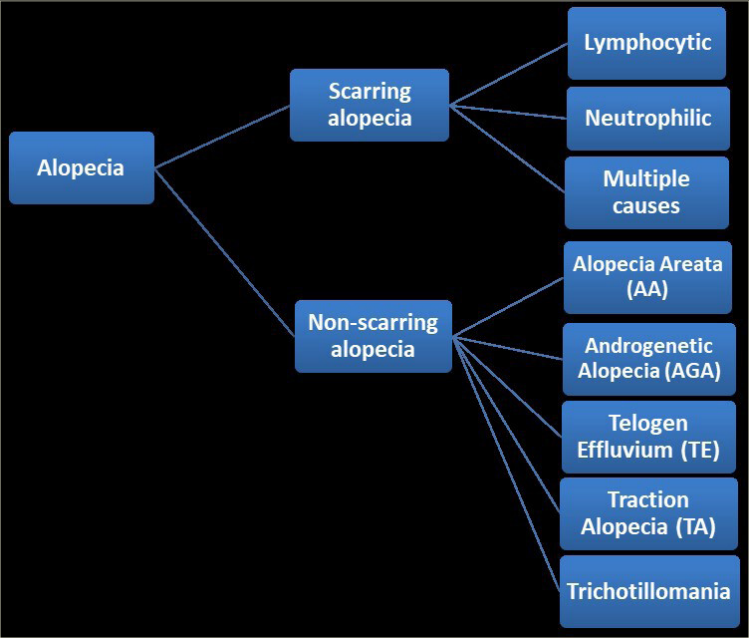
Types of alopecia

Scarring alopecia is further subdivided into lymphocytic, neutrophilic and multiple causes (*Figure 1*).^1,3^

### Lymphocytic primary scarring alopecia

Discoid lupus erythematosusDiscoid lupus erythematosus can occur in the absence of any systemic disease or in conjunction with systemic lupus erythematosus.^9,12–14^ It is known to progress with exposure to sunlight (ultraviolet light), so it mainly affects the scalp, face and ears, causing scarring alopecia and facial disfigurement.^6,12–14^ The clinical features of discoid lupus erythematosus include alopecia characterized by well-defined, coin-shaped persistent erythematous indurated plaques of varying size, followed by follicular hyperkeratosis, which is skin adherent.^5,9,12–14^ When the adhering scale is removed, follicle-sized keratotic spikes like carpet tacks can be observed (“carpet tack sign”).^5,9,12–14^ The lesions slowly extend with active inflammation and hyperpigmentation at the periphery, resulting in depressed central atrophy, scarring, telangiectasia and hypopigmentation. Discoid lupus erythematosus can lead to permanent scarring alopecia on the scalp.^5,9,12–14^

#### Keratosis follicularis spinulosa decalvans

Keratosis follicularis spinulosa decalvans is a rare genetic disorder that often starts in infancy or early childhood and predominantly affects males.^9,15–18^ It presents with progressive scarring alopecia of the scalp and eyebrows.^9,15–18^

#### Lichen planopilaris

Lichen planopilaris is characterized by a lymphocytic invasion of hair follicles and is common in women between the ages of 40 and 60.^19–21^ Patients with lichen planopilaris may experience itching, burning or tenderness of the scalp.^19,20^

#### Frontal fibrosing alopecia

Frontal fibrosing alopecia is an irreversible chronic lymphocytic scarring alopecia; its cause is unknown.^22,23^ It is distinguished by progressive regression of the frontal and temporal hairlines, usually associated with loss of eyebrows and eyelashes.^9,22–24^

#### Alopecia mucinosa

Alopecia mucinosa refers to permanent hair loss that occurs when hair follicles are replaced by mucin.^7,9,25^ Clinically, it presents as erythematous-infiltrated plaques with follicular prominence, especially in the head and neck.^25^

#### Central centrifugal scarring alopecia

Central centrifugal scarring alopecia (CCSA) typically manifests as irreversible hair loss on the crown of the scalp and progresses in a centrifugal pattern to the parietal scalp.^5,9,26,27^ The exact cause of CCSA is unknown and considered to be multifactorial. A gene variant was recently discovered in approximately 25% of patients with CCSA; however, the exact role of this variant in the occurrence of CCSA is unclear.^26–28^

#### Pseudopelade of Brocq

Pseudopelade of Brocq is a rare, chronic and gradually progressive form of scarring alopecia that mainly affects middle-aged women.^8,29^ The vertex and parietal scalp are commonly involved, and it is distinguished by the clinical appearance of small skin-coloured alopecia patches that look similar to "footprints in the snow", and mild-to-moderate atrophy with no evidence of folliculitis or significant inflammation.^7–9,29^

### Neutrophilic primary scarring alopecia

#### Dissecting cellulitis of the scalp, or Hoffman's disease

Dissecting cellulitis of the scalp, or Hoffman's disease, is a rare form of neutrophilic alopecia of unknown aetiology that leads to irreversible hair loss.^30–32^ The clinical features of dissecting cellulitis of the scalp are nodules, abscesses on the vertex and posterior scalp, draining sinuses and scarring alopecia.^30–32^

#### Folliculitis decalvans

Folliculitis decalvans is the most common type of neutrophilic alopecia.^33–35^ It is characterized by painful follicular pustules that expand centrifugally, erosions and scaly-crusty lesions localized in the scalp, which leave central scarring and hair loss.^33–35^ Folliculitis decalvans typically affects only the scalp; however, one report has mentioned the involvement of the limbs and trunk.^34^

### Mixed primary scarring alopecia

#### Acne keloidalis nuchae

Acne keloidalis nuchae is a chronic inflammatory form of scarring folliculitis.^36–40^ The clinical features are the occurrence of keloid-l ike papules, pustules and plaques; it most commonly affects the occipital scalp and posterior neck.^36,39^ The exact aetiology of acne keloidalis nuchae is unclear. However, this condition may be caused by chronic mechanical irritation to the back of the neck and scalp, trauma, heat, humidity, infection, autoimmune disorder, medications, and excess of some hormones such as androgens.^36–38^ Treatment, which aims to improve the symptoms of the disease, is difficult and unsatisfactory; many treatments have been tried with varying degrees of success.^38^

#### Acne necrotica

Acne necrotica is a bizarre and enigmatic rare follicular disorder that manifests as a necrotizing disorder of the hair follicle.^7,9,40^ It is characterized by the development of papules on the scalp with central necrosis in adult patients and causes pox-l ike scars.^7,9^ It represents a diagnostic challenge due to its distinct clinical pathological features.^40^

#### Erosive pustular dermatosis of the scalp

Erosive pustular dermatosis of the scalp is a rare chronic inflammatory skin disease that causes scarring alopecia and mainly affects older adults.^41–43^ The condition is commonly found on the scalp or legs but can occur anywhere on the skin.^41^ Clinical diagnosis may be difficult because it is similar to more common diseases, such as squamous cell carcinoma, infection, localized scarring pemphigoid (the Brunsting-Perry type), or folliculitis decalvans, and has non-specific clinical characteristics (crusts, atrophy, and pustules).^41–43^

## Non-scarring alopecia

Non-scarring alopecia, also called non-cicatricial alopecia, refers to recoverable hair loss.^1,3,44^ It is classified into androgenetic alopecia (AGA), telogen effluvium (TE), traction alopecia, trichotillomania and alopecia areata (AA) (*Figure 1*).^1,3,44^

### Androgenetic alopecia

AGA, often called male pattern baldness, is an autosomal dominant disorder that gradually transforms terminal hairs into intermediate and vellus hairs.^45–47^ It is the most common progressive baldness in both men and women; however, clinical symptoms differ between genders. In men, AGA causes gradual thinning and vertex loss; in women, hair loss affects the frontal scalp and vertex, while the frontal hairline is spared, resulting in a more visible scalp.^45,47^ Testosterone metabolite and dihydrotestosterone are the most important mediators of AGA. They act on androgen receptors in the hair follicles, causing hair thinning in the anagen phase and extending the telogen phase of hair growth, leading to more immature hair and decreasing the new hair.^45–47^ Some studies linked AGA to systemic diseases such as metabolic syndrome, endocrine diseases, mental disorders and hypothyroidism.^45,46^ The treatment for AGA consists of oral finasteride, topical external use of minoxidil, low-intensity laser and hair transplantation; however, whether the treatments are successful remains controversial.^45–47^

### Telogen effluvium

TE is a common non-scarring alopecia that mainly affects women.^47–50^ It consists of diffuse hair loss occurring 2–3 months after a triggering event and lasting about 6 months; this hair loss leads to an abnormal shift of scalp hair follicles from anagen to telogen, resulting in premature hair shedding.^47–49^ TE can be caused by low ferritin, vitamin B12 deficiency, thyroid dysfunctions, systemic diseases, drugs, medications, fever, stress, weight loss and giving birth.^47–50^

### Traction alopecia

Traction alopecia occurs in individuals who have hairstyles that exert a consistent pulling force on the hair roots.^51,52^ It is most common in women of African descent, who may braid their hair tightly and use chemical treatments to straighten it.^51,52^ In the early stages, patients with traction alopecia usually present with patches of non-scarring hair loss and tensioned areas of the scalp; in its later stages, the disease can progress to irreversible scarring alopecia if left untreated.^51^ Patients may also report tenderness, itching and headaches.^51^

### Trichotillomania

Trichotillomania is a psychiatric condition classified as an obsessive– compulsive disorder, in which a person pulls out hair from any part of their body regularly, resulting in hair loss.^53–55^ It can affect any part of the body; however, it most commonly affects the scalp, followed by the eyebrows and the pubic region.^53–55^ Clinically, it appears as patches of varying size and shape; however, the patches are not completely bald but are covered in an irregular pattern of broken hair.^47^

### Alopecia areata

AA is the most frequent cause of inflammation-induced, immune-mediated non-scarring hair loss disorder.^56–58^ It has an unpredictable course and a broad spectrum of manifestations (*Figure 1*).

Although it is uncommon in children under 3 years, the vast majority of patients with AA are young. Up to 66% of patients are under 30, and only 20% of patients are older than 40.^56–62^ AA is characterized by a rapid onset, with patients experiencing hair loss in well-circumscribed patches, usually round or oval in shape, and entirely hairless and smooth.^56,58,59,61^ In 90% of clinical diagnoses, AA's most common clinical feature is a circumscribed area of bald skin on the scalp that is usually isolated from other patches.^57,58,60^ Since AA is a dynamic disorder (i.e. causing recurrent episodes of hair loss), patches may appear in other parts of the body, such as the elbows, arms and thighs.^60^ Furthermore, the disease may affect facial hair, including eyelashes, eyebrows and the beard area.^60^ One of the factors that may influence the onset of AA is stress.^47,57,62–65^ Some patients reported an emotional event or an identity crisis prior to the onset of AA.^47,57,62–65^ Other factors, such as infections, toxins, genetic factors and even food, could be triggers of the disease. AA is often associated with other diseases, such as systemic lupus erythematosus, autoimmune haemolytic anaemia, asthma, atopic dermatitis, vitiligo, allergic rhinitis, and thyroid diseases.^47,57,62–65^

## Thyroid autoimmune diseases and alopecia

Several known TADs cause hypothyroidism (e.g. Hashimoto's thyroiditis [HT], painless thyroiditis and postpartum thyroiditis), hyperthyroidism (e.g. Graves’ disease [GD]), or subacute thyroiditis (e.g. De Quervain thyroiditis).^66^ The most common TADs are GD and HT.^67^ TADs, which are also marked by autoantibodies targeting thyroid follicular cells, are the most common autoimmune disease associated with alopecia.^68^

HT is the most common cause of autoimmune hypothyroidism.^67,69^ Women are more affected by HT than men, with a ratio of 18:1; however, men are increasingly affected by this pathology.^70^ One of the most common clinical signs of HT is alopecia.^68–70^ GD is the most common hyperthyroidism, resulting from the hyperstimulation of the thyroid gland due to the presence of thyrotropin receptor antibodies that bind and activate the thyroid-stimulating hormone receptor, increasing synthesis of triiodothyronine (T3) and the prohormone thyroxine (T4).^71^ GD is an autoimmune disease associated with AA.^72,73^ Thyroid hormones are involved in hair growth, and alopecia affecting different areas of the body is one of the clinical signs of thyroid dysfunction.^74^ The presence of alopecia and thyroid dysfunction increases with age.^74^ Moreover, androgenic alopecia, the most common form of alopecia affecting men and women of all ages, is strongly correlated with autoimmune thyroiditis, especially hypothyroidism.^75–78^ Due to the high prevalence of hair loss and thyroid dysfunction, exploring the thyroid function is recommended in patients with alopecia.^79^

It was reported that more than 42.7% of patients with AA express thyroid autoantibodies such as anti-thyrotropin receptor antibodies, suggesting that anti-thyroid and anti-hair follicle autoimmunity share a similar aetiology.^72^ Furthermore, the prevalence of thyroid antibodies was found to be significantly associated with AA.^80,81^ Genetic variability between some populations might also affect the frequency of TAD. The frequency of AA varies geographically; for instance, in the Korean population, thyroid diseases, including hyperthyroidism, hypothyroidism, goitre and thyroiditis, were the most common in late-onset AA.^82^

AA is an autoimmune disease that targets the hair follicles by T cell-mediated autoimmune reaction.^83,84^ The aetiology of AA is not well understood; however, it is strongly associated with thyroid dysfunction such as HT.^85^ Several studies showed a high prevalence of thyroid autoimmunity (GD and HT and various thyroid disorders) associated with AA.^80–85^ A 3-year retrospective study by Chen et al. also found a high prevalence of AA in TAD.^86^ The occurrence of thyroid diseases such as HT, endemic goitre and GD in patients with AA ranges between 8% and 28%.^87^ AA is mostly associated with GD and with the presence of thyrotropin receptor antibodies.^72^

Alopecia is associated with other autoimmune diseases, such as vitiligo, coeliac disease, diabetes mellitus, psoriasis and lupus erythematous.^88^ It may also be associated with genetic predisposition and stress.^89^ Stress can increase P (SP)-i mmunoreactive nerve fibres, leading to mast degranulation and activation of pro-inflammatory cytokines and chemokines in the thyroid gland.^83,90^ Similarly to TAD, alopecia was found to have an hereditary component: it was linked significantly to human leukocyte antigen Class II alleles DR4, DR11, DPw4, DQw3, DQw7, and DQw8 and to HLA Class I alleles A28, B12, B13, B18, and B27.^87^ AA shares a similar mechanism with TAD, characterized by the involvement of the CD8+ and CD4+ T cells targeting hair follicle auto-a ntigens resulting in cytokines production.^88^ Cytokine interferon (IFN-) is the most important factor produced in AA via CD4+ Th1-mediated response.^83^ The pro-inflammatory cytokine interleukin-1 (IL-1) recruits inflammatory cells such as T-l ymphocytes, neutrophils and macrophages to the affected tissue. Together with tumour necrosis factor (TNF)-alpha, they suppress hair growth.^91^ TNF, together with other Th1 cytokines (IFN, IL-2 and IL-12), are increased in lesion biopsies of patients with AA, with TNF being the most important factor.^92^ The lesion is inflammation induced and reversible as it causes a non-scaring loss of hair follicles.^90^ Moreover, the infiltration of hair follicles by the T lymphocytes results in the release of other factors, such as TNFs and enzymes, causing apoptosis of the cells in the hair follicle.^93^

## The link between thyroid hormones and hair follicles

Insight into the hair growth process reveals that every single hair on the scalp passes through four stages: the anagen phase, in which the hair grows from the follicle; the catagen phase, in which the follicle shrinks, and the hair detaches from the scalp; the telogen phase, in which a new hair begins to grow underneath the old hair; and the exogen phase, wherein the old hair falls off and is replaced by the new hair.^94^ As part of this process, old hairs at the end of their growth cycle constantly fall out from the scalp, causing the loss of approximately 50–100 hairs per day. However, since the hair follicles do not all grow synchronously, a daily loss does not impact the physical appearance.^94^ Understanding this simple mechanism is crucial to understanding how thyroid disorders can affect the induction of alopecia and chronic hair loss.

The structure and function of the hair and skin can certainly be impacted by low or high levels of thyroid hormones, such as the active hormone T3 and the prohormone T4, the main product of the thyroid gland.^95^ However, the specific mechanism by which T3/T4 stimulation affects human hair follicles, and whether an increase or decrease in T3 and/ or T4 levels directly influences the development of alopecia, are yet to be known. Thyroid hormones play a crucial role in regulating myriads of cellular functions, including growth, differentiation, metabolism and thermogenesis.^95,96^ Hence, disruption of the production of thyroid hormones, especially T3 and T4 hormones, can affect many essential bodily processes, including the healthy growth of hair. Thyroid hormones may contribute to hair loss by interfering with the body's normal hair development cycle. If the thyroid function is damaged, the hair might not grow as fast as it normally would, so when old hairs get to the end of their hair growth cycle, new ones do not sufficiently replace them. Hence, thyroid disorders cause alopecia. As the evidence indicates, both T3 and T4 sustain the length of the hair development phase (anagen) *in vitro*, which might be the result of the downregulation of the important anagen-inhibitory growth factor (TGF-2) by thyroid hormones.^95,96^

As hair growth is a highly energy-c onsuming process,^97^ Vidal et al. hypothesized that thyroid hormones promote mitochondrial function and regulate human energy metabolism in hair follicles, which leads to an increase in the expression of some mitochondrial genes and proteins that regulate intra-follicular mitochondrial energy metabolism and the redox status of human hair follicles.^97,98^ The results of these studies collectively indicated that thyroid hormones directly target hair follicles and dramatically affect their biology. Therefore, hair loss can occur when these hormones are produced abnormally. However, the molecular mechanisms have not yet been fully understood. Both thyrotropinreleasing hormone and its receptor are expressed in the human scalp hair follicles.^99^ As a result, it is possible that the hypothalamic–pituitary– thyroid pathway exists outside of the hair follicles in humans.

## Thyroid cancer and alopecia

The first study to describe alopecia following thyroid cancer therapy was published in 1998.^100^ The authors believed that the amount of radiation exposure to hair follicles is much below the level required to result in hair loss. Therefore, the temporary, non-dose-dependent occurrence of hair loss cannot be a result of the radioiodine therapy, and it is most likely the outcome of thyroid hormone status changes due to thyroid cancer.

Thyroid hormone receptors, which are crucial for controlling the hair's growth cycle, have been found in hair follicles.^101^ Moreover, the assumption that thyroid cancer causes alopecia and *vice versa* is based on hormone involvement in AA and on the fact that some thyroid diseases are linked to alopecia.^102^ It has been shown that Taiwanese women suffering from alopecia are more likely to develop thyroid cancer than those without alopecia.^103^ While structural thyroid abnormalities did not appear to be associated specifically with any type of alopecia,^104^ the risk of thyroid cancer is increased based on the alopecia subtype.^105^ A previous study of 12,199 patients with AA found that a significant increase in the risk of thyroid cancer was observed in patients with AA; however, a significant decrease was noted in the risk of other types of cancers, such as breast, colon/rectum, stomach, liver and lung cancer.^106^ Moreover, it is hypothesized that alopecia might inhibit carcinogenesis due to its immunological and genetic similarities to multiple sclerosis, whose innate immune profile (characterized by increased T helper type 1 immune activation and altered cytokine levels) reduces the risk of developing cancer.^107^ In addition, the correlation between alopecia and thyroid cancer was investigated and proved in the presence of two TADs (GD and HT).^103,108–112^ Moreover, patients with AA were found to be at greater risk of TAD,^113^ and alopecia was found to be significantly associated with thyroid cancer.^113–115^ A recently published study in *Cancer Epidemiology* described a 26-year-old female patient who was diagnosed with thyroid cancer 8 years before developing alopecia.^116^

Except for thyroid cancer, which is more common in people with alopecia, the correlations between solid organ tumour cancers and alopecia have been negligible.^116,117^

## Conclusions

In this focused review of articles published between 2011 and 2022, we showed that alopecia is correlated with TAD and thyroid cancer.

Thyroid hormones contribute to hair loss by interfering with the normal cycle of hair development; if the thyroid function is damaged, the hair takes longer and requires more energy to regrow. Therefore, alopecia is an effect of thyroid disorders. However, the specific mechanism by which the production of thyroid hormones, especially T3 and T4 hormones, affects human hair follicles and the development of alopecia, is still unknown.^94–96^

Moreover, hypothyroidism, in which the thyroid gland does not produce enough thyroid hormones, or hyperthyroidism, in which the thyroid gland produces excess thyroxine hormone, often result from autoimmune conditions. Many of these patients have a higher risk of developing autoimmune conditions such as alopecia, which causes patches of hair loss in discrete areas leading to baldness. With that established, it is easier to see why thyroid conditions can cause alopecia.^66–79^ AA is an autoimmune disease targeting the hair follicles through a T cell-mediated autoimmune reaction that is strongly correlated with thyroid dysfunction.^83–85^ The exact cause of alopecia is not well known, but AA is strongly associated with TAD.^80–85^

Similarly, thyroid cancer and alopecia have been significantly associated. The direct action of thyroid hormone has physiological effects on the scalp and hair follicles resulting in alopecia.^107,113–115^ Although a significant correlation between thyroid cancer and TAD was found in patients with alopecia, the relationship is mostly negligible and needs to be investigated further.

In conclusion, the literature demonstrates complete understanding of alopecia and its types. However, more studies are required to fully understand the mechanism and association between TAD and alopecia, as well as between alopecia and thyroid cancer.
